# Tuberculosis–Cancer Parallels in Immune Response Regulation

**DOI:** 10.3390/ijms21176136

**Published:** 2020-08-26

**Authors:** Thomas E. Bickett, Sana D. Karam

**Affiliations:** Department of Radiation/Oncology, University of Colorado Anschutz Medical Campus, Aurora, CO 80022, USA; thomas.bickett@cuanschutz.edu

**Keywords:** immunotherapy, *Mycobacterium tuberculosis*, radiation therapy, tumor immunology

## Abstract

*Mycobacterium tuberculosis* and cancer are two diseases with proclivity for the development of resistance to the host immune system. Mechanisms behind resistance can be host derived or disease mediated, but they usually depend on the balance of pro-inflammatory to anti-inflammatory immune signals. Immunotherapies have been the focus of efforts to shift that balance and drive the response required for diseases eradication. The immune response to tuberculosis has widely been thought to be T cell dependent, with the majority of research focused on T cell responses. However, the past decade has seen greater recognition of the importance of the innate immune response, highlighting factors such as trained innate immunity and macrophage polarization to mycobacterial clearance. At the same time, there has been a renaissance of immunotherapy treatments for cancer since the first checkpoint inhibitor passed clinical trials, in addition to work highlighting the importance of innate immune responses to cancer. However, there is still much to learn about host-derived responses and the development of resistance to new cancer therapies. This review examines the similarities between the immune responses to cancer and tuberculosis with the hope that their commonalities will facilitate research collaboration and discovery.

## 1. Introduction

**Figure 1:**
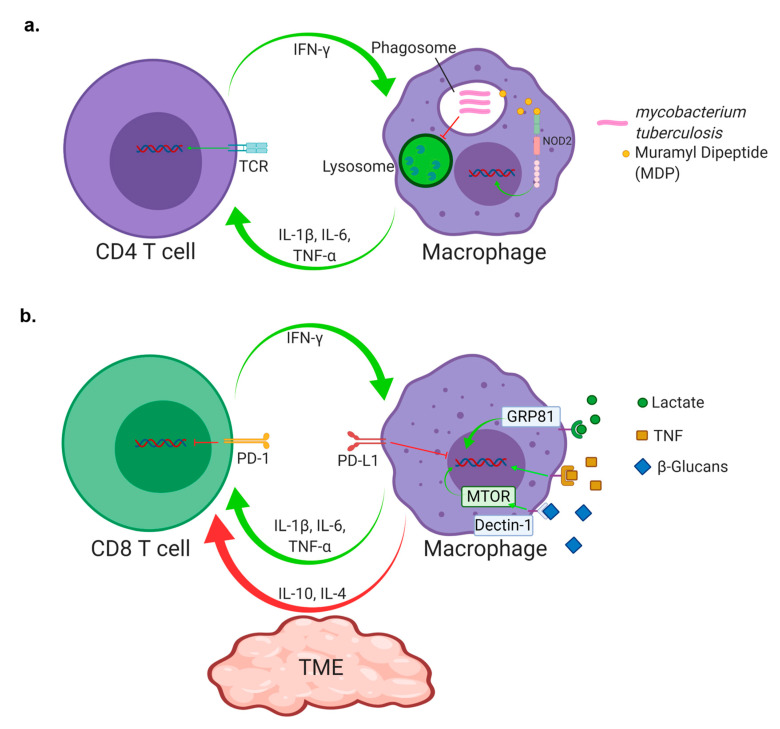
(**a**) The killing of mycobacterium within a phagosome of a macrophage is mediated by IFN-γ production from CD4 T cells. *Mycobacterium tuberculosis* prevents the fusion of the phagosome and lysosome and can persist inside a macrophage for extended periods. The release of muramyl dipeptide (MDP) by M. tuberculosis is critical for activation of the NOD2 receptor and instigating trained innate immunity; (**b**) CD8 T cells, which are key for the development of a proper anti-tumor response, are heavily reliant on cytokine input from macrophages. Innate immune training takes place in the tumor microenvironment (TME) via macrophage stimulation by β-glucans, and lactate. Macrophages are also heavily regulated by TNF stimulation. Additionally, checkpoint inhibition through the programmed cell death protein 1/programmed death ligand 1 (PD-1/PD-L1) pathway is a currently used technique by which the interaction between CD8 T cells and macrophages can be modified by immunotherapy.

Both *Mycobacterium tuberculosis* (Mtb) and cancer involve processes that reflect a highly evolved and coordinated program of immune evasion strategies that interfere with innate and adaptive immunity. Central to tuberculosis granulomas and the tumor microenvironment (TME) is a common theme of a hijacked macrophage polarization program and an exhausted T cell phenotype that contribute to resistance mechanisms of these diseases. In both tuberculosis and cancer, initial detection by macrophages is a crucial step that primes the immune response. When *M. tuberculosis* is inhaled into the lung, it is endocytosed and sequestered in the phagosome of an alveolar macrophage [1]. Strong stimulation is required by T cells for macrophage activation and subsequent mycobacterial killing [2] (Figure 1a). *M. tuberculosis* has evolved special mechanisms for surviving these processes in detoxifying the toxic chemicals before harm is done [3,4,5,6], or in repairing the damage before it becomes lethal [7,8,9]. In addition, *M. tuberculosis* is known to escape the phagosome and persist in the cytoplasm of the macrophage (Figure 1a). This is what leads to the hallmark of tuberculosis (TB) disease, the granuloma [10,11] (Table 1). Granuloma formation is a complex process involving immune evasion of the mycobacterium through the aforementioned mechanisms and continued interaction between infected macrophages and T cells. During this time, mycobacterial growth slows to where the population of bacteria is thought to be sustained by non-replicating bacteria inside macrophages [12,13]. This latency in growth permits persistent inflammation during which the surrounding tissue becomes more vascularized, allowing for increased macrophage and T cell recruitment [14]. At the infection site, macrophage cells differentiate into a variety of subclasses. Macrophages are critical for granuloma formation because of their response mechanisms in inflammation and tissue repair [15,16]. Layers of the granuloma become more defined as a fibrous cuff forms outside the stratum of macrophages. Finally, lymphocytes form follicle-like centers surrounding the fibrous cuff, which walls off infected macrophages and the necrotic center where the mycobacteria are trapped [17]. This period of infection is known as latency, and the infection progresses no further. The immunological processes behind latency are dependent on T cell–macrophage interaction, as a lack of macrophage activation would lead to uncontrolled mycobacterial growth [18], yet the immune suppression required is also macrophage mediated. Classically activated macrophages, or M1 macrophages, drive inflammation, whereas alternatively activated macrophages, or M2 macrophages, are anti-inflammatory and contribute to wound healing [19].

Similarly, from an early stage, the TME is heavily influenced by T cell–macrophage interactions. Myeloid cells comprise up to 50% of the leukocytes within a tumor, with the majority of these being macrophages [26]. Similarly to tuberculosis, M2 macrophages play a role in preventing the immune system from eliminating disease. Immunosuppressive macrophage populations are known as tumor-associated macrophages (TAMs) and are responsible for much of the anti-inflammatory signals in the TME [27]. TAMs can arise from either tissue-resident macrophages or circulating monocytes, but these populations are maintained by a steady influx of circulating monocytes [26]. As a result of their association with wound healing, macrophages are poised to assist in angiogenesis for tissue growth. TAMs are also known to suppress T cell responses [28]. Here, we also see an important interaction between macrophages and T cells that can influence the overall outcome of disease. This review focuses on immune evasion in TB and cancer as it relates to macrophage and T cells to explore how it might apply to future immunotherapy development. In particular, we focus on immunologically cold tumors, as a lack of immune activation is the greatest obstacle. We would to acknowledge that the literature is too vast to cover in complete detail, but we will attempt to summarize here some of the more recent and salient observations in the field.

## 2. Macrophages: Potent Modulators of Protection from the Innate Immune System through Trained Innate Immunity

A large portion of TB literature focuses on vaccine development. Bacille Calmette Guerine or BCG, a live attenuated strain of *Mycobacterium bovis*, is the only preventive vaccine against TB used to vaccinate infants worldwide [29] and represents one of the oldest cancer immunotherapeutic agents [29,30,31]. How BCG functions remains poorly understood, but its ability to activate an innate immune system, particularly macrophages, explains its ability to act as a preventive and therapeutic vaccine in TB and cancer, respectively. Unfortunately, BCG does not protect against pulmonary tuberculosis infection in humans, and any protection established against disseminated tuberculosis infection wanes into adolescence [32]. The reasons for this are unclear, but geographic location has been identified as a factor where efficacy increases along with distance from the equator [33]. It is possible that this is due to environmental exposure to mycobacteria, but more evidence is needed. However, in rodents, BCG is able to induce protection against tuberculosis in addition to protection against non-mycobacterial diseases [34]. Subcutaneous BCG vaccination also protects against *M. tuberculosis* infection within 7 days, in a process independent of T cells [35]. As a potent biological response modifier, BCG is capable of modulating the innate immune system, particularly macrophages, to resist infection [36].

Recent work has introduced the concept of trained innate immunity, which is a mechanism that allows the innate immune system to possess a memory-like response and is typically an attribute assigned to adaptive immunity [35,36]. Trained innate immunity is dependent on the NOD2 receptor, a cytosolic pathogen recognition receptor (PRR) that recognizes muramyl dipeptide (MDP) from bacterial peptidoglycan [37] (Figure 1a). Upon NOD2 binding of MDP, epigenetic reprogramming allows that particular cell to “remember” the exposure to MDP. Further stimulation with MDP triggers the NOD2 receptor to elicit a more rapid and robust response featuring increased pro-inflammatory cytokine release including IL-1β, IL-6, and TNF-α. This has been proposed as an explanation for the ability of BCG to induce non-specific protection not only to infection from *M. tuberculosis*, but other non-mycobacterial infections [35,36,38] (Figure 1a). Although originally identified in monocytes, other cells such as NK cells can also undergo innate immune training [39]. Mechanisms such as innate immune training offer insight into how BCG functions in the induction of immunity. However, much remains unknown. Although BCG does induce trained innate immunity, separate uncharacterized mechanisms may be responsible for the protection observed in the mouse model of tuberculosis. An induction of trained innate immunity is dependent on the NOD receptor [36]. However, other work shows that BCG-mediated protection through innate immunity can still occur in the absence of the NOD receptors. Such protection is thought to be mediated by hyperactivation of the innate immune system, including the recruitment of activated macrophages into the lung after subcutaneous vaccination [35]. This ability of a subcutaneous BCG vaccination to induce the recruitment of monocytes to the lung within seven days adds to its immunotherapeutic potential.

As trained innate immunity influences macrophage polarization, it has potential benefits in anti-tumor immunity. Within the TME, macrophage populations tend to favor alternative polarization, or an M2 phenotype. Such polarization is characterized by high IL-10 production and low IL-12 production, keeping the TME in an anti-inflammatory state [19,40,41]. The preexisting hypoxic conditions within solid tumors can contribute to the production of IL-1β, which can lead to the production of VEGF, promoting angiogenesis and M2 polarization, and enhancing tumor growth [42]. Cancer fibrosis is also induced through the production of TGF-β1 by M2 macrophages, rendering tumors more difficult to treat [43]. As macrophage polarization states show a high degree of plasticity and can change with external stimuli, macrophage “training” can be therapeutically leveraged to exert an anti-tumoral effect within the TME. Regulating the polarization of macrophages from M2 to M1 phenotype is an important step in limiting the pro-tumor effect of TAMs [44].

As trained innate immunity is independent of T cells, it is important to distinguish this macrophage-mediated BCG response from the delayed hypersensitivity reaction similarly brought on by BCG. While delayed hypersensitivity is initiated by mononuclear leukocytes, it culminates with a strong T cell response 24–72 h later. In fact, BCG has been used in the past as an immunostimulant adjuvant along with tumor antigen in a vaccine study for colon cancer [45]. Although tumor-specific T cells were identified in patients after vaccination, these vaccines never panned out, as tumor-specific T cells are not enough on their own for tumor eradication [46]. Indeed, it seems that the interaction of BCG with other immune cell types may be critical for better defining the role of BCG in vaccination against TB and cancer.

## 3. Innate Immune-Based Immunotherapies for Cancer and TB

BCG has been used in the past as a treatment for bladder cancer that works through potent stimulation of the innate immune system. The activation of neutrophils and production of neutrophil extracellular traps (NETs) can directly lead to tumor cell kill [47]. The direct stimulation of macrophages with BCG can also elicit high levels of TNF-α that can result in direct tumor cell kill [48]. Importantly, this leads to the recruitment of T cells and production of IFN-γ, IL-2, and TNF-α, which leads to tumor cell kill, but not necessarily through tumor specific mechanisms [49]. Although these mechanisms require local BCG stimulation to induce direct cancer-killing properties, a strong conversion from a Th2-like response to a Th1 immune response has also been observed in responders to intravesicle BCG therapy, suggesting that, similar to TB [50], BCG-mediated changes in polarization of the immune system are protective against cancer, and they are more important for the immune response than the induction of antigen-specific T cells [51]. Therefore, the proper training of macrophages represents a way to reverse the negative effects of immune polarization in the TME, with BCG as an example, as a method for this training. It is known that in certain cancers, such as head and neck squamous cell carcinoma (HNSCC), the increased presence of M2-polarized macrophages correlates with negative clinical prognostic markers [52]. Although cancer is not a natural source of MDP to induce trained innate immunity, there is evidence to suggest that artificially stimulating epigenetic reprogramming within the TME can induce anti-cancer immune responses. The induction of trained innate immunity with muramyl tripeptide in patients with osteosarcoma has shown success by increasing overall and disease-free survival [53]. Others have identified β-glucans from yeast as a potential method of inducing trained innate immunity in the TME through the pathogen recognition receptor (PRR) dectin-1 [25] (Figure 1b). This process of trained innate immunity is dependent on the activation of mTOR pathways to epigenetically modify the cells [54]. This is particularly pertinent to antitumor immunity, as other work has identified that cancer cell-induced training of innate immune cells can occur through the mTOR pathway (Figure 1b). It is hypothesized that this is induced through the production of lactate by the cancer cell that interacts with the lactate receptor GPR81 on monocytes [55]. Such epigenetic reprogramming can assist in keeping TAMs activated and pro-inflammatory [55] (Figure 1b). It is unknown how artificially stimulating this same pathway with β-glucans can be utilized for immunotherapy. Although not considered trained innate immunity, polarization switching from M2 to M1 has been achieved through CD40 agonists [21], the use of β-glucan from yeast [22], and in STAT3 inhibitors [23] (Table 1). Perhaps macrophage training induce by BCG or other sources should be considered as an alternative.

Conversely, both CD40 agonists and STAT3 inhibitors have shown potential for translation into tuberculosis research. CD40 agonists have been observed to boost the T cell response to TB infection [56], but they have not been evaluated for inducing the classical activation of macrophages. Similarly, studies have shown that STAT3 activation does correlate to impaired T cell function in tuberculosis patients [57]. Additionally, the formation of foamy macrophages has been linked to STAT3, suggesting that STAT3 inhibitors could potentially play a role in macrophage polarization in tuberculosis [58]. Other immunotherapeutic approaches against TB have come from an unlikely source. Recent studies have highlighted the potential that rifampicin, in addition to being an effective antibiotic to treat tuberculosis, might be an effective immunotherapeutic. Early studies of human lung epithelial cells showed that rifampicin can increase the production of nitric oxide synthase (iNOS) [24]. Current understanding shows that rifampicin can increase the amount of iNOS and therefore nitric oxide (NO) produced by macrophages in response to pro-inflammatory cytokines such as IL-1β, IFN-γ, and TNF-α. NO production serves as an important mediator of host defense to tuberculosis [59] and an important cytokine for classifying M1 polarized macrophages [60]. Although studies are ongoing, rifampicin is, in theory, an excellent combination antibiotic and immunotherapeutic for the treatment of tuberculosis.

## 4. Checkpoint Inhibition

Checkpoint inhibitors represent a growing area of research in cancer immunotherapy, and its applicability potentially extends to tuberculosis. In the TME, T cell exhaustion leads to ineffective immune responses, and the associated molecular mechanisms have been reviewed elsewhere [61]. In tumor immunotherapy, successful treatment methods so far have relied on immune checkpoint inhibitors with targets such as programmed cell death protein 1 (PD-1), programmed death ligand 1 (PD-L1), and cytotoxic T-lymphocyte associated protein 4 (CTLA-4). The underlying premise is that the controlled downregulation of regulatory immune pathways can lead to increased efficacy of the immune system’s clearance of an infection or tumor [62,63]. Chronic infections such as tuberculosis are similar to cancer in that there is an extended period of time with high levels of antigen exposure. Prolonged exposure to tuberculosis antigens does lead to an increased expression of PD-1 on CD4 and CD8 T cells, and blocking PD-1 or PD-L1 with an antibody increases CD4 and CD8 T cell activity [64]. PD-1 blockade also shows some promise in saving effector T cells from apoptosis during pulmonary infection [65]. As T cell exhaustion has been shown to restrict the functionality of ESAT-6-specific CD4 T cells during *M. tuberculosis* infection [66], it is reasonable to think from a mechanistic standpoint that anti-exhaustion therapies will improve the immune response to tuberculosis. While mechanistically sound, this has unfortunately not proven to be the case [67]. Evidence suggests that the blockade of PD-1/PD-L1 is not enough to rescue effector T cell function in the case of chronic *M. tuberculosis* infection. In addition, mice lacking the PD-1 receptor (PD-1^−/−^) show increased susceptibility to pulmonary tuberculosis [68,69]. It is hypothesized that this is due to a lack of homeostasis in the immune response and subsequent immune hyperactivation leading to inflammatory damage. Furthermore, PD-1 expressing T cells during *M. tuberculosis* infection are not exhausted T cells in the classical sense, as they actually play a role in maintaining antigen-specific effector T cells [70]. Epigenetic remodeling that accompanies T cell exhaustion is also not affected by PD-L1 blockade and can lead to a relapse of exhaustion if antigen presence remains high. This leads to exhausted populations failing to convert to memory responses [20]. Tuberculosis is not alone in lack of a response to PD-1 blockade; for example, HNSCC has shown response rates as low as 10–15% [71,72]. As such, there is a need for alternative immunotherapies to reverse T cell exhaustion in diseases such as tuberculosis and HNSCC through the use of other checkpoint inhibition molecules. T cell immunoglobulin and mucin domain-containing-3 (TIM3) is another immunotherapy target that shows promise. Unlike with PD-L1, the blockade of TIM3 actually does improve outcomes to *M. tuberculosis* infection, and this is thought to be largely mediated through the restoration of exhausted T cells [73,74]. Furthermore, it has been identified that PD-1 blockade actually increases TIM3 expression in HNSCC. Combination approaches with a dual blockade of both PD-1 and TIM3 may be required to overcome T cell exhaustion in both of these diseases [75].

## 5. T Cell Infiltration and Homing

Proper homing of T cells has been identified as a major problem within the TME of multiple cancer types [76]. In lung squamous cell carcinoma, the lack of T cell infiltration into tumors is a poor prognostic indicator. In this particular example, imaging clearly shows the presence of an abundance of T cells gathered on the periphery of tumor islets, but a lack of infiltration [77]. The interaction between TAMs and the CD8 T cells within the tumor stroma has been reported as a main barrier that prevents intratumoral T cell infiltration [77]. Although targeting macrophages may represent a crucial first step in identifying new mechanisms to improve intratumoral T cell homing in cancer immunotherapies [77], it is important to note that the blockade of macrophages through CSF-1R in lung cancer models increased T cell infiltration, but not tumor clearance. Antitumor responses were only improved upon the addition of anti-PD-1 [77]. This highlights the importance of T cell activation and the role for macrophages in T cell homing. Cytotoxic T Lymphocyte Associated protein 4 (CAR-T) cell therapy is also benefiting from increasing T cell homing through CXCL2-related mechanisms [78].

Immunotherapies to facilitate proper T cell homing also have a potential to aid the treatment of tuberculosis [79]. Although BCG can function in the mouse model independent of T cells, most vaccine development for *M. tuberculosis* is centered on an induction of strong T cell responses in the lungs following vaccination to induce long-term protection [80]. Upon infection in the lung, antigen-presenting cells (APCs) are responsible for trafficking to the mediastinal lymph node to activate *M. tuberculosis*-specific CD4^+^ T cells [81,82]. Following T cell priming and clonal expansion, T cells circulate to the lungs. These T cells are part of the Th1 immune response, which is dependent on cytokines such as IL-12 and IFN-γ (Table 1). IL-12 produced by APCs induces the expression of T-bet, which is a T cell transcription factor that is known for further activating the Th1 response [83]. In addition to its key role in activating macrophages [84], IFN-γ also plays a role in inducing further T-bet expression [85]. However, upon arrival to the infected lung tissue, many T cells are unable to cross to the lung parenchyma and instead home to the lung vasculature, where they are non-protective [86]. Although these vascular T cells produce higher levels of IFN-γ, they are less protective than the populations of T cells that home into the parenchyma. This is even maintained after adoptive transfer of the parenchymal populations [87]. From this, it has been hypothesized that although IFN-γ plays an important role in mycobacterial killing in the spleen, it may not be as important for protection from tuberculosis in the lung [88]. Further evidence has shown that this lack of killing may be due to the inability of T cells to localize to the center of the granuloma where infected macrophages reside [89]. Future T cell-based immunotherapies for cancer and tuberculosis would be aided by accounting for not only T cell activation, but also T cell homing.

## 6. T Cell Antigen Exposure and Exhaustion

Antigen exposure in the TME is currently an area of high focus due to modern techniques and the availability of newly generated datasets. Recent studies have identified that durable antitumor immune responses require the activation of both CD8 and CD4 T cells through MHC-I and MHC-II molecules, respectively [90]. Multiple cancer types including colorectal, melanoma, head and neck, and lung cancers have demonstrated good clinical outcomes in correlation with a strong TH1 immune response [91,92] and a strong memory T cell response being a driving factor behind lasting antitumor immunity [93]. The role of T cells in antitumor immunity has been heavily tied to the identification of neoantigens, which are antigens recognized by CD4 and CD8 T cells that are unique enough so as to not get confused with self-recognizing T cells. Neoantigen discovery has an important role in the future of tumor immunity, as T cells specific to neoantigens not only improve the overall TME in immunologically cold tumors [94], but they are also less susceptible to immune tolerance [95]. Neoantigen discovery has the potential to benefit new CAR-T cell therapy by improving the cell targets. Additionally, in conjunction with a wide variety of proteomic and genomic techniques, cancer vaccines against tumor mutations, even specific to the mutanome of individual patients, are options being explored for cancer vaccines [94,95,96,97,98,99]. In particular, recent advancements with the single-cell characterization of T cells can assist in identifying specific T cell populations for therapeutic intervention [100]. Although these methods are reliant on T cells, their relevance to tuberculosis remains low, as neoantigens are a tumor-specific phenomenon, and options for anti-cancer vaccines have been extensively reviewed elsewhere [96].

The induction of antigen-specific T cell responses has posed unique problems for designing efficacious vaccines for *Mycobacterium tuberculosis*. The importance of both CD4^+^ and CD8^+^ T cells to survival after *M. tuberculosis* infection has been demonstrated in both the mouse and non-human primate model of tuberculosis [101,102,103,104]. However, vaccine-induced T cells have not been so easily linked to increased protection. For example, after BCG vaccination, IFN-γ-producing T cells specific to mycobacterial antigen do not always correlate to a reduction in mycobacterial burden [105]. Recent evidence suggests that T cells specific for tuberculosis antigens face contradictory complications when attempting to clear infection; specifically, with two common vaccine target antigens Ag85B and ESAT-6. Ag85B is a protein that is not heavily expressed during active disease, whereas ESAT-6 is highly expressed during that time. T cells corresponding to these antigens can be found in the lungs of vaccinated mice. Unfortunately, T cells specific to both of these antigens show very limited protection to *M. tuberculosis* infection for very unique reasons, as *Mycobacterium tuberculosis* undergoes different cycles of growth during infection. During active disease, when the mycobacterium is actively replicating, AG85B is expressed in very low levels. Ag85B-specific T cells show a limited control of infection due to these low levels of antigen expression. Alternatively, ESAT-6 is a highly expressed protein during active infection. Specific T cells to ESAT-6 are prone to over activation and T cell exhaustion [66]. Proper antigen exposure due to replication cycles is an important component of immunotherapy design and timing of treatment. The application of new single-cell technologies to identify problematic T cell populations would greatly aid therapeutic intervention.

## 7. Discussion

Although the two diseases are immunologically similar, the research conducted in the fields of tuberculosis and cancer have separate focuses. Tuberculosis research has centered on prevention, while cancer research has mostly focused on immune-mediated clearance of an already established disease. Given the parallels between the immune responses to these diseases, the hopes of this review are to bridge the two fields, so that some of the vast literature of cancer research is usable in the tuberculosis field, and the niche immunology knowledge gained in tuberculosis research is available to the cancer field. As tuberculosis research has uncovered, there are many factors that can influence the efficacy of immunotherapies, including antigen availability during disease progression and the fact that PD-1 may not be an exhaustion-specific marker that is useful for efficient checkpoint inhibition. There is also the potential of BCG as a future biological response modifier affecting macrophage polarization. Future immunotherapies for cancer can consider this for the timing of treatment and potential combination therapies. The enormous volume of cancer work has revealed much for the field of tuberculosis; checkpoint inhibition through a combination of PD-1 and TIM3 may be required for the successful reversal of T cell exhaustion, and macrophage polarization immunotherapies have been successfully used to modulate the immune response. Given that macrophage polarization is thought to be responsible for BCG-induced protection, other means of modulating macrophage polarization merit consideration. If these diseases are to be cured, a strong collaboration between these two fields will be key in finding new angles and therapies.

## Figures and Tables

**Table 1:** T cells and macrophages are key players in both the anti-cancer immune response and tuberculosis. Their roles, the cytokines involved, and potential immunotherapies are briefly summarized here.

**Summary Table**		
**T Cells**		
	**Tuberculosis**	**TME**
**Role**	Activation of M. tuberculosis infected macrophages to stimulate mycobacterial killing Possess an effector role that has potential to be modified through immunotherapy [2]	Elimination of Cancer Cells Immunomodulation of the TME with an effector role that has been modified through immunotherapy [20]
**Cytokines**	IFNγ, TNF-α, IL-6, IL-12, IL-1β	
**Immunotherapies**	Anti-PD-1/PD-L1	Anti-PD-1/PD-L1, CAR T cells, TIGIT, OX40, 4-1BB, LAG3, TIM-3, Monalizumab, CTLA-4
**Macrophages**		
	**Tuberculosis**	**TME**
**Role**	The main reservoir for M. tuberculosis Work in conjunction with T cells to kill mycobacteria [2]	Immunomodulatory roles through cytokine production Effector role that can be modified through immunotherapy [21,22,23]
**Cytokines**	iNOS, IFNγ, TNF-α, IL-6, IL-1β, IL-10, IL-4, IL-2	
**Immunotherapies**	CD40 agonists [23] Rifampicin [24]	CD40 agonists [23] β-Glucan [25] STAT3 [22]

## Abbreviations

BCG	Bacille Calmetter Guerine
CTLA-4	Cytotoxic T Lymphocyte Associated protein 4
HNSCC	Head and Neck Squamous Cell Carcinoma
MDP	Muramyl Dipeptide
NETs	Neutrophil Extracellular Traps
NO	Nitric Oxide
PD-1	Programmed Death Protein 1
PD-L1	Programmed Death Protein Ligand 1
TAM	Tumor Associated Macrophage
TB	Tuberculosis
TIM-3	T cell Immunoglobulin and Mucin Domain Containing-3
TME	Tumor Microenvironment
Mtb	*Mycobacterium tuberculosis*
